# Robot-Assisted versus Laparoscopic Partial Nephrectomy for Giant Sporadic Renal Angiomyolipomas of ≥7 cm: A Propensity Score-Matched Analysis

**DOI:** 10.1155/2021/6395876

**Published:** 2021-08-26

**Authors:** Yunqiang Xiong, Wen Deng, Ru Chen, Xiaoqiang Liu, Ke Zhu, Jiayao Wang, Jiahui Long, Haoxin Jiang, Luyao Chen, Bin Fu

**Affiliations:** ^1^Department of Urology, The First Affiliated Hospital of Nanchang University, Yongwai Street 17, Nanchang City, Jiangxi Province, China; ^2^Department of Urology, The First Hospital of Putian City, Putian City, Fujian Province, China

## Abstract

**Background:**

To compare the perioperative and functional outcomes between robot-assisted partial nephrectomy (RAPN) and laparoscopic partial nephrectomy (LPN) for giant sporadic renal angiomyolipomas (AMLs) of ≥7 cm.

**Materials and Methods:**

Patients with sporadic renal AMLs of ≥7 cm who underwent RAPN or LPN in the First Affiliated Hospital of Nanchang University between 2015 and 2020 were retrospectively analyzed. Propensity score matching (1 : 1) was performed to adjust for potential baseline confounders. Perioperative and functional outcomes of the RAPN and LPN groups were collected and compared.

**Result:**

After propensity score matching, no statistically significant differences in baseline characteristics were found between the groups (41 vs. 41). Within the matched cohort, the warm ischemia time (WIT) in the RAPN group was significantly shorter than that in the LPN group (21 vs. 27 min, *p* < 0.001). In addition, the RAPN group was associated with improved postoperative renal function (72.8 vs. 69.8 mL/min/1.73 m^2^, *p*=0.045). WIT and preoperative renal function are independent predictors of renal function at 6 months postoperatively, and renal score and operation method are independent predictors of WIT.

**Conclusion:**

RAPN and LPN are safe and feasible minimally invasive treatments for sporadic giant renal AMLs, but RAPN is associated with shorter WIT and better postoperative renal functional preservation. WIT and preoperative renal function are independent predictors of renal function at 6 months postoperatively, while the RENAL score and surgical method are independent risk factors to WIT. For giant and complex renal AMLs, RAPN is the first choice when condition permits.

## 1. Introduction

Renal AML is a benign mesangial mesenchymal tumor that originated from renal chylous cells [1]. Its incidence is about 0.4% in the general population, accounting for 3% of all renal tumors [2]. It is composed of fat, smooth muscle, and blood vessels and is prone to rupture and bleeding. Most AMLs are sporadic, accounting for about 80%; the remaining 20% are found at the time of diagnosis of tuberous sclerosis [3]. Sporadic AMLs are common in women and are often unilateral and slow growing [4]. Treatment depends on size (traditionally, renal AMLs larger than 4 cm are recommended for aggressive treatment [5]), presence of symptoms, and pregnancy status and should be tailored to the patient with the goal of renal function [6].

At present, the main means of AMLs treatment include selective arterial embolization (SAE), nephron sparing surgery (NSS) (including minimally invasive surgery and open surgery), and ablation, which includes a wide range of modalities but is used infrequently [7]. The major complication of AMLs is retroperitoneal hemorrhage because of tumor rupture, which may affect the safety of the patient's life. SAE is the first-line treatment for AMLs hemorrhage, but it is prone to recurrence, with 30% of cases requiring a second operation [8]. Although NSS is more destructive than SAE, it has the huge advantage of having a lower risk of recurrence. Recurrence of AMLs after NSS is rare, occurring in only 0–3% of cases [9]. Minimally invasive surgical procedures, such as robot-assisted laparoscopic partial nephrectomy (RAPN) and laparoscopic partial nephrectomy (LPN), are commonly employed. Open surgery is rarely used because of its many disadvantages, such as serious injuries, serious bleeding, and slow recovery [10].

Whether or not RAPN and LPN differ in efficacy in AMLs treatment remains unclear. Several studies focused on the treatment of AMLs of ≥4 cm RAPN versus LPN [11, 12]. Partial resection of giant renal AMLs is a difficult surgery, and a specific study on renal AMLs of ≥7 cm remains lacking to date.

## 2. Materials and Methods

We retrospectively analyzed the data of all patients bearing AMLs treated using RAPN or LPN in the First Affiliated Hospital of Nanchang University between 2015 and 2020 with the approval of the institutional review board and ethics committee of our institution. Inclusion criterion was as follows: renal AML of ≥7 cm (≥7 cm was identified as giant in this paper). Exclusion criteria were as follows: bilateral or multiple tumors and cases with a confirmed tuberous sclerosis, missing important data, postoperative pathology other than renal AML, off-clamp NSS, within the learning curve of surgeons. A total of 93 patient data were obtained. This study was approved by the institutional ethics committee of our hospital, and informed consent was obtained from all patients included in the data. All patients underwent preoperative abdominal CT or MRI examination. Baseline measures included gender, age, tumor location, body mass index (BMI), tumor size, RENAL score, preoperative glomerular filtration rate (GFR), symptoms, underlying disease, history of abdominal surgery, and previous history of SAE. Study measures included warm ischemia time (WIT), estimated intraoperative blood loss, time to complete surgery, postoperative hospital stay, postoperative complications, and GFR at 6 months postoperatively. RENAL scores were calculated from preoperative imaging (preferred CT), and all scores were performed by the same clinically experienced surgeon. With the exception of GFR at 6 months postoperatively, all data were obtained during the patient's surgical hospitalization.

### 2.1. Surgery

All surgeries were performed by experienced urological surgeons. The retroperitoneal or transabdominal transperitoneal approach was used according to the surgeon's preference. Intraoperative renal ischemia was achieved by clamping the renal artery. The tumor was removed by cutting the tumor 0.5 cm along the edge of the tumor or attracting with a super large aspirator. Some patients received SAE preoperatively, and the distance from NSS was 3 months or longer. Frozen sections were sent before intraoperative resection of the tumor.

### 2.2. Measurement and Statistical Analysis

A propensity score matching analysis was performed to control for selection bias and confounding factors. On the basis of the estimated propensity score, the patients treated with RAPN and those treated with LPN were matched using the 1 : 1 approach in the matching strategy, with no substitution.

Independent-sample *t*-test, Pearson chi-square test, or nonparametric rank-sum test was used to compare the covariate differences before and after matching. Matching enhanced the balance between the two process groups. The medical records and surgical results were compared and analyzed. The operative results included complete operative time, WIT, intraoperative estimated blood loss, postoperative complications, and postoperative hospital stay. GFR was measured 6 months after surgery. Average and standard error reports were used for continuous variables with normal distribution. Median and percentile values were reported for nonnormally distributed numerical variables, and proportion was reported for categorical variables. Comparison tests (classified variable chi-square test and continuous variable Mann–Whitney test) were used to evaluate the difference in surgical outcomes between patients receiving RAPN and LPN. Regression analysis was performed to predict the influence of dependent variables. Statistical test was double-tailed test, and bilateral *p* ≤ 0.05 indicated statistical significance. SPSS26.0 software was used for statistical analysis.

## 3. Results

In total, 52 patients received LPN and 41 patients received RAPN. The baseline demographic and clinical characteristics are shown in Table 1. Before the propensity score matching, a statistical difference in renal score was found between the RAPN and LPN groups (9 vs. 8, *p*=0.01). After 1 : 1 matching, 41 cases remained in each group. The balance of the key features was tested, and the results showed that all the matching features reached a good balance among the matching groups. No significant differences in gender (*p*=0.448), age (*p*=0.429), tumor location (*p*=0.184), BMI (*p*=0.973), symptoms (*p*=0.073, *p*=0.09, *p*=0.364), underlying disease (*p*=1, *p*=0.305), history of abdominal surgery (*p*=1), mean tumor size (*p*=0.693), RENAL score (*p*=0.297), and preoperative GFR (*p*=0.396) were found among all groups (all *p* > 0.05). During the follow-up period, no recurrence occurred in either group.

The perioperative data and renal function at 6 months postoperatively are shown in Table 2. In the matched cohorts, no significant differences in complete operative time (175 vs. 190 min), estimated intraoperative blood loss (200 vs. 200 mL), postoperative hospital stay (7 vs. 7 day), and complications (2.4% vs. 7.3%) were found between the RAPN and LPN groups. The RAPN group had significantly shorter WIT than the LPN group (21.0 vs. 27.7 min, *p* < 0.001). The number of cases exceeding 25 min in the LPN group was also significantly higher than that in the RAPN group (61.0% vs. 4.9%, *p* < 0.001). In terms of renal function at 6 months postoperatively, the two groups (72.76 vs. 69.83 mL/min/1.73 m^2^, *p*=0.045) also had a significant difference (renal function of all patients was greater than 60 mL/min/1.73 m^2^ at 6 months postoperatively).

A linear regression analysis of renal function at 6 months postoperatively is shown in Table 3. Single-factor screening showed that age (*p* < 0.001), maximum tumor diameter (*p*=0.001), operation type (*p*=0.045), WIT (*p* < 0.001), and preoperative GFR (*p* < 0.001) affected renal function at 6 months postoperatively. Multiple linear regression showed that WIT (*p*=0.003) and preoperative GFR (*p* < 0.001) are independent predictors of renal function at 6 months postoperatively.

We continued to perform linear regression analysis on WIT, and the results are shown in Table 4. Univariate screening revealed that RENAL score (*p*=0.003) and operating method (*p* < 0.001) were the factors that may affect WIT. Multivariate linear regression analysis showed that RENAL score (*p* < 0.001) and operating method (*p* < 0.001) are independent predictors of WIT.

## 4. Discussion

With the development of surgical technology and the improvement of people's quality of life, the minimally invasive technique of preserving nephron has been widely used in urological surgery. On the basis of a large number of retrospective studies on SAE and NSS, NSS has the advantages of complete tumor resection and lower recurrence rate [9, 13, 14]. When patients require intervention, NSS may be a better option. Since the advent of the da Vinci robotic surgical system, the comparison between RAPN and LPN has become a “hot” issue. Compared with LPN, RAPN performs better perioperatively because of its clearer field of view, more flexible angle, and more stable operation [15]. However, most studies about RAPN vs. LPN were based on renal AMLs with a diameter of more than 4 cm [11, 12], and no studies specifically focused on renal AML with a diameter of ≥7 cm. Indisputably, partial resection of giant AMLs is one of the difficulties in urological surgery. Thus, a research about the efficacy of RAPN vs. LPN in treating giant renal AMLs is necessary.

In our research, we obtained some important results. On the basis of good matching between the RAPN and LPN groups (Table 1), Table 2 shows that WIT (*p* < 0.001) and GFR at 6 months postoperatively (*p*=0.045) were significantly different between the RAPN and LPN groups. Subgroup analysis revealed that WIT (*p*=0.003) and preoperative GFR (*p* < 0.001) are independent predictors of renal function at 6 months postoperatively (Table 3). The RENAL score (*p* < 0.001) and operating method (*p* < 0.001) are independent predictors of WIT (Table 4). Same as other studies [16, 17], no significant differences were found between the two groups in terms of complete operation time, intraoperative estimated blood loss, postoperative hospital stay, and postoperative complications (Table 2).

WIT is an important indicator of perioperative period [18]. Kidney is a highly oxygen-dependent organ, and the damage to kidney caused by WIT is mainly caused by ischemia reperfusion [19], which directly affects the postoperative renal function of patients. At present, the minimum safe range of WIT remains controversial. Up to now, only one prospective study has timely performed renal needle biopsy in 40 patients at 30 min after renal ischemia, and the analysis has suggested that a 30–60 min WIT period during which minor structural changes occur in the renal system without severe functional loss may be safe [20]. Hot ischemia (>25–30 min) may also cause irreversible ischemic damage to the surgically treated kidney [21]. Each additional minute of renal ischemia increases the risk of acute kidney injury by 5%–6%, leading to severe chronic kidney disease [22]. However, 25 min is within the undetermined safe range of WIT, and the shorter the time of WIT, the more beneficial it is to the renal function of patients. In the present study, the WIT of the RAPN group was significantly shorter than that of the LPN group (21 min vs. 27 min, *p*=0.001). This result is largely attributed to the Da Vinci robot's advantages of 3D vision, flexible and accurate operation, and fast stitching speed [15]. A study reported that the mean WIT of LPN in the treatment of renal AMLs (in 20 patients, the mean tumor size was 6.4 cm) is 25.3 min [23]. RAPN has also been reported in the treatment of renal AMLs (in 53 patients, the mean tumor size was 2.8 cm, and the RENAL score was 6), with a mean WIT of only 17.5 min [24]. In other similar studies, the WIT was generally shorter than ours under the same operation method. The size of the tumor and the difficulty of the surgery are important reasons. In recent years, the application of off-clamp technique in minimally invasive partial nephrectomy has attracted extensive attention [25–27]. Several studies have demonstrated that off-clamp technique was safe and feasible and offered better postoperative renal function preservation than traditional on-clamp technique, especially in RAPN [28–31]. It is noteworthy that off-clamp surgery is technically demanding, with potential for increased blood loss, and requires considerable experience with partial nephrectomy surgery. In our center, off-clamp technique has been attempted to apply in some giant AML cases received RAPN (data not shown in present manuscript). Future clinical studies involving larger datasets and long-term follow-up are needed to further explore the impact of off-clamp technique on giant and complex AMLs.

The purpose of NSS is to preserve the renal function of patients in the long term on the basis of complete tumor resection to improve the quality of life of patients [32]. Control studies of RAPN vs. LPN for treating AMLs are few and far between. Our study demonstrated that the patients treated with RAPN were superior to those treated with LPN in terms of renal function at 6 months postoperatively (*p*=0.045). This result can be ascribed to several possible reasons. First, giant renal AMLs are large in size and complex in anatomical structure, and robotic systems have a clearer field of view and more flexible operation, allowing more renal parenchyma to be preserved during tumor resection. In addition, long-term renal function is highly correlated with the number of intraoperative retained nephroids [33]. Second, Rod et al. [34] believed that irreversible damage to renal function would occur if the WIT exceeds 25 min. The average WIT of the two procedures was 21 vs. 27 min (*p* < 0.001), respectively, and the number of cases exceeding 25 min in the LPN group was also significantly higher than that in the RAPN group (61.0% vs. 4.9%, *p* < 0.001).

Table 3 shows that WIT and preoperative GFR are independent predictors of renal function at 6 months postoperatively. A retrospective study of 226 patients by Zargar et al. suggested that tumor size, preoperative estimate GFR, and WIT are important predictors of long-term renal function [35]. This result is close to our conclusion. The more nephrons preserved, the better the preoperative GFR, and the better the long-term renal function will be after surgery. When faced with patients with giant renal AMLs, urologists must shorten the WIT as much as possible intraoperatively. Table 4 demonstrates that the operating method and RENAL score are independent predictors of WIT. Therefore, when the tumor is larger and more complex, PAPN can result in shorter WIT and better long-term renal function than LPN.

Clinically, partial resection of giant renal AMLs after a certain period of SAE (e.g., 3 months) is also an option. Unfortunately, only five patients in our study had SAE preoperatively, indicating that this procedure is unsuitable for this study. However, to date, whether or not using NSS directly after SAE in treating AMLs differs in efficacy has not been demonstrated. One study [36] that included only 36 patients concluded that the application of SAE before LPN can decrease the difficulty of the surgery, the complications, and the risk of rebleeding. Additional studies are needed to provide evidence.

This study has some limitations. This study has a nonrandomized retrospective design. Key variables such as age, BMI, comorbidities, tumor size, RENAL score, and renal function were matched, but some potential selection bias or confounding factors were possibly not controlled. Renal function at 6 months postoperatively is not exactly equivalent to long-term renal function. In addition, the samples were all from a single medical center, and further multicenter randomized controlled trials are needed. Despite these limitations, this study is the largest series of studies on giant AMLs to date. More large, prospective, randomized studies are needed to validate our findings.

## 5. Conclusion

In our study, both robot-assisted and laparoscopic PN are safe and feasible treatments for sporadic renal AMLs of ≥7 cm, but robot-assisted PN was associated with shorter warm ischemia time and better postoperative renal functional preservation when compared with laparoscopic PN. Both the WIT and preoperative renal function are independent predictors of renal function at 6 months postoperatively, while the RENAL score and surgical method are independent risk factors to WIT. For giant and more complex renal AMLs, RAPN is the first choice when condition permits.

## Figures and Tables

**Table 1:** Preoperative characteristics by surgery type before and after propensity score matching.

Variables	Before propensity score matching			After propensity score matching		
	RAPN (*n* = 41)	LPN (*n* = 52)	*p*	RAPN (*n* = 41)	LPN (*n* = 41)	*p*
Gender (female), *n* (%)	32 (78.0%)	33 (63.5%)	0.324	32 (78.0%)	29 (70.7%)	0.448
Age (years), mean (SD)	41.7 (12.4)	43.6 (1.03)	0.498	41.7 (12.4)	43.7 (10.6)	0.429
BMI (kg/m^2^), mean (SD)	23.2 (1.06)	23.2 (1.02)	0.963	23.1 (1.05)	23.1 (0.90)	0.973
Mean tumor size (cm), median (IQR)	8.2 (7.5, 9.6)	8.2 (7.5, 9.5)	0.804	8.2 (7.5, 9.6)	8.1 (7.5, 9.3)	0.693
Left tumor, *n* (%)	25 (61.1%)	27 (51.9%)	0.946	25 (61.1%)	19 (46.3%)	0.184
RENAL score, median (IQR)	9 (8, 10)	8 (7, 9)	0.01	9 (8, 10)	9 (8, 9)	0.297
Symptoms						
Lumbage, *n* (%)	28 (68.3%)	26 (50.0%)	0.964	28 (68.3%)	20 (48.8%)	0.073
Hematuresis, *n* (%)	5 (12.2%)	2 (3.8%)	0.13	5 (12.2%)	1 (2.4%)	0.09
Tumor rupture, *n* (%)	5 (12.2%)	9 (17.3%)	0.494	5 (12.2%)	8 (19.5%)	0.364

Underlying disease						
Hypertension, *n* (%)	5 (12.2%)	6 (11.5%)	0.922	5 (12.2%)	5 (12.2%)	1
Diabetes, *n* (%)	3 (7.3%)	2 (3.8%)	0.922	3 (7.3%)	1 (2.4%)	0.305

Abdominal surgery history, *n* (%)	7 (17.1%)	9 (17.3%)	0.976	7 (17.1%)	7 (17.1%)	1
Preoperative SAE, *n* (%)	2 (4.9%)	6 (11.5%)	0.255	2 (4.9%)	4 (9.8%)	0.396
GFR (mL/min/1.73 m^2^), mean (SD)	82.38 (5.63)	82.61 (5.71)	0.832	82.38 (5.63)	82.51 (5.62)	0.906

SD: standard deviation; IQR: interquartile range; BMI: body mass index; AML: angiomyolipoma; GFR: glomerular filtration rate.

**Table 2:** Perioperative indicators and GFR at 6 months postoperatively.

Variables	RAPN (*n* = 41)	LPN (*n* = 41)	*p*
Complete operation time (min), median (IQR)	175 (145, 245)	190 (147.5, 227.5)	0.82
WIT (min), mean (SD)	21.02 (3.17)	27.73 (7.98)	<0.001
>25 min, *n* (%)	2 (4.9%)	25 (61.0%)	<0.001
Intraoperative blood loss (ml), median (IQR)	200 (100, 225)	200 (100, 400)	0.122
Postoperative hospital stay (days), median (IQR)	7 (6, 8.5)	7 (6, 9)	0.57
Complication (*n*), *n* (%)	1 (2.4%)	3 (7.3%)	0.305
Postoperative GFR (mL/min/1.73 m^2^), mean (SD)	72.76 (6.34)	69.83 (6.91)	0.045

SD: standard deviation; IQR: interquartile range; WIT: warm ischemia time; GFR: glomerular filtration rate.

**Table 3:** Renal function at 6 months postoperatively of linear regression analysis.

Variables	Single-factor screening		Multiple-factor analysis	
	*B*	*p*	*B* (95% CI)	*p*
Gender	0.26	0.263		
Age	−0.49	<0.001	−0.08 (−0.18, 0.01)	0.072
BMI	−0.67	0.390		
Mean tumor size	−0.37	0.001	−0.10 (−0.66, 0.45)	0.714
RENAL score	−0.10	0.356		
Lumbage	−0.16	0.150		
Hematuresis	0	1		
Tumor rupture	−0.12	0.268		
Hypertension	−0.15	0.180		
Diabetes	−0.14	0.210		
Abdominal surgery history	0.05	0.688		
Operating method	0.22	0.045	−1.34 (−3.4, 0.80)	0.214
Complete operation time	−0.04	0.743		
WIT	−0.49	<0.001	−0.25 (−0.41, −0.09)	0.003
Intraoperative blood loss	−0.15	0.166		
Postoperative hospital stay	−0.05	0.648		
Preoperative GFR	0.71	<0.001	0.73 (0.48, 0.98)	<0.001
Preoperative SAE	−0.10	0.374		

BMI: body mass index; WIT: warm ischemia time; GFR: glomerular filtration rate.

**Table 4:** WIT of linear regression analysis.

Variables	Single-factor screening		Multiple-factor analysis	
	*B*	*p*	*B* (95% CI)	*p*
Gender	−0.07	0.969		
Age	0.08	0.214		
BMI	0.74	0.356		
Mean tumor size	0.38	0.361		
RENAL score	2.21	0.003	2.59 (1.40, 3.78)	<0.001
Lumbage	1.50	0.336		
Hematuresis	0.49	0.868		
Tumor rupture	2.76	0.190		
Hypertension	0.48	0.838		
Diabetes	0.14	0.970		
Abdominal surgery history	0.06	0.976		
Operating method	6.71	<0.001	7.28 (4.85, 9.70)	<0.001
Estimate intraoperative blood loss	0.001	0.597		
Preoperative SAE	2.90	0.328		

WIT: warm ischemia time; BMI: body mass index; SAE: selective arterial embolization.

## Data Availability

The datasets generated for this study are available from the corresponding author upon request.
